# ISOLATED VERSUS SUPPORTED: THE PERSONAL HEALTH CARE NETWORKS OF OLDER ADULTS LIVING WITH TYPE 2 DIABETES

**DOI:** 10.1093/geroni/igae098.0803

**Published:** 2024-12-31

**Authors:** Catherine Clair, Karen Bandeen-Roche, Jennifer Wolff, Janiece Taylor, Katherine Ornstein, Karin Tobin

**Affiliations:** Johns Hopkins Bloomberg School of Public Health, Baltimore, Maryland, United States; Johns Hopkins University, Baltimore, Maryland, United States; Johns Hopkins University, Baltimore, Maryland, United States; Johns Hopkins University, Johns Hopkins University, Maryland, United States; Johns Hopkins University, Baltimore, Maryland, United States; Johns Hopkins University, Baltimore, Maryland, United States

## Abstract

Older adults with Type 2 diabetes may or may not have family and friends involved in their care; using social network methods, we can investigate the personal healthcare networks of this population. Objectives included 1) Describe care networks of older adults with Type 2 diabetes; and 2) Characterize the family and friends who provide support for various activities of daily living (ADL), instrumental activities of daily living (IADL), and medically-oriented tasks. We administered a quantitative ego-centric social network inventory to community-dwelling older adults (65+) living with Type 2 diabetes (n=58). The sample was on average, 73.1 years old (SD: 5.9, Range: [65, 86]). and majority female (n=44, 75.9%) and non-White (n=37, 63.8%). Descriptive statistics were conducted overall and by Black (n=32) and White (n=21) race. Ten (17.5%) participants reported no family or friends involved in their care; six were Black and four were White. Those experiencing care isolation were more likely to have never been married compared to those with care networks (p=0.002). Of those with a network, there was an average of 2.9 (SD: 2.0, Range: [1, 11]) individuals in the network; there were no statistically significant differences in network size by race. Of the 140 family and friends across the entire sample, 23 (16.4%) assisted with ADLs, 126 (90.0%) with IADLs, and 48 (34.3%) with medically-oriented tasks. Fourteen (10.0%) individuals assisted with at least 1 activity in all three categories. These findings demonstrate a spectrum of family and friend involvement in the care of this population.

